# An Integrated Framework for Multi-State Driver Monitoring Using Heterogeneous Loss and Attention-Based Feature Decoupling

**DOI:** 10.3390/s22197415

**Published:** 2022-09-29

**Authors:** Zhongxu Hu, Yiran Zhang, Yang Xing, Qinghua Li, Chen Lv

**Affiliations:** 1 School of Mechanical and Aerospace Engineering, Nanyang Technological University, Singapore 639798, Singapore; 2 Centre for Autonomous and Cyber-Physical Systems, Cranfield University, Bedford MK43 0AL, UK; 3 Alibaba DAMO Academy Autonomous Driving Lab, Hangzhou 311121, China

**Keywords:** driver state, feature decoupling, cascade cross-entropy, gaze consistency

## Abstract

Multi-state driver monitoring is a key technique in building human-centric intelligent driving systems. This paper presents an integrated visual-based multi-state driver monitoring framework that incorporates head rotation, gaze, blinking, and yawning. To solve the challenge of head pose and gaze estimation, this paper proposes a unified network architecture that tackles these estimations as soft classification tasks. A feature decoupling module was developed to decouple the extracted features from different axis domains. Furthermore, a cascade cross-entropy was designed to restrict large deviations during the training phase, which was combined with the other features to form a heterogeneous loss function. In addition, gaze consistency was used to optimize its estimation, which also informed the model architecture design of the gaze estimation task. Finally, the proposed method was verified on several widely used benchmark datasets. Comprehensive experiments were conducted to evaluate the proposed method and the experimental results showed that the proposed method could achieve a state-of-the-art performance compared to other methods.

## 1. Introduction

**Figure 1:**
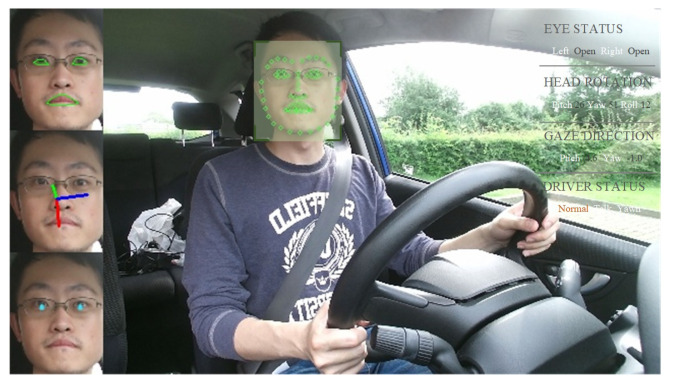
Example of our multi-state driver monitoring system.

Intelligent agents are now part of our lives and their numbers are expected to increase [1,2,3]. Therefore, the smartness, safety, and efficiency of interactions and collaborations between humans and agents should continue to improve [4,5,6,7,8,9]. In particular, autonomous driving technology could allow humans to share control with intelligent vehicles [10,11,12,13,14,15]. A well-designed co-pilot system requires the vehicle to understand the behavior and state of the driver [16,17,18,19,20,21]. Owing to the low costs and wide application of dash cameras, this paper proposes an integrated multi-state driver monitoring framework based on appearance, which does not use intrusive sensors. The framework incorporates several common functions, such as head rotation, gaze, blinking, and yawning, as shown in Figure 1. For convenience, our design principles include the following:

1. The different modules should be independent of each other so the functions are convenient to use;
2. The framework should be easy to maintain and update based on the development of deep learning technology;
3. The framework should provide various configuration models to coordinate computing power and accuracy;
4. The included functional modules should achieve state-of-the-art performance.

In particular, head pose estimation (HPE) and 3D gaze estimation (GE) are important and challenging indicators of a driver’s state and attention. This paper focuses on these two factors and discusses them in depth. The other modules are briefly described.

Generally, there are two types of methods that are used to estimate these two factors: geometry-based [22] and appearance-based methods [23,24]. Geometry-based methods rely on prior models and have high requirements for the quality and resolution of the input images. Advances in deep neural networks (DNNs) have focused considerable attention on the use of appearance-based models in a data-driven manner. Researchers have proposed different types of DNN-based structures to achieve improved performance. Neural architecture search (NAS) technology provides general structures and backbones, such as the EfficientNet series [25]. In order to follow principles 2 and 3, this study focused on leveraging the prior knowledge of tasks to maximize the effects of the proposed backbone model.

In current research, DNN models are well known for classification tasks [26]. Therefore, this study converted HPE and GE into classification tasks to improve the performance of the model. There are no correlations between different classes in general classification tasks, but this would be unreasonable for the HPE and GE tasks that were converted from regression tasks. To handle this problem, a heterogeneous loss was introduced, which included the proposed cascade cross-entropy and maintained the continuity of the estimations. For the architecture of the proposed model, an open pre-trained model was used as the feature extractor, i.e., EfficientNet. In addition, this study assumed that the different angles of GE and HPE were independently distributed. A feature decoupling module (FDM) was developed to decouple the extracted features from different axis domains by leveraging a channel attention module. The HPE and GE could apply similar strategies, but the important difference was that the GE could use left-eye images and right-eye images simultaneously. Usually, the gaze directions of the left and right eyes are consistent; therefore, this study proposed the principle of gaze consistency. Based on this principle, the predictions of the left and right eyes could be optimized.

This paper presents the following three main contributions: an integrated appearance-based multi-state driver monitoring framework, which could be used to build human-centric intelligent driving systems; a unified network architecture combined with a heterogeneous loss function for HPE and GE that could improve the performance of the model and outperform other state-of-the-art methods; the principle of gaze consistency significantly optimizing gaze estimation.

The paper is organized as follows. Section 2 comprehensively discusses related work. Section 3 describes the proposed network architecture, the training loss function, and the gaze consistency principle. Section 4 analyzes and discusses multiple experimental results. Then, the conclusions and future work are presented in Section 5.

## 2. Related Work

This study mainly focused on visual sensor-based driver posture estimation.

### 2.1. Visual Sensor-Based Head Pose Estimation

Head pose estimation has been widely studied using various methods and different modal sensors, including infrared sensors and depth sensors. Most of these studies used RGB images as the input, as in this study.

From the task perspective, HPE can be addressed as landmark-free or landmark-based learning using facial landmarks. The landmark-based strategy hypothesizes that the position of facial landmarks is related to head pose. Many approaches simultaneously estimate head pose and conduct other face-related tasks using the same network. Some approaches even derive head pose directly from facial landmarks [22,27]. While this is theoretically reasonable, but these methods are not usually robust and cannot achieve the best performance. This is because landmark-based methods require the ground truth facial landmarks and head pose to be very precise, as demonstrated in [27], in which the ground truth was relabeled to achieve a better performance than using the original label. Several landmark-free approaches have achieved state-of-the-art performances on open datasets. HOPE-Net is a representative method in this field [28,29] and is an elegant and robust approach to detecting head pose using a dual-loss network, which inspired our work. FSA-Net is a hierarchical classification approach for soft stage-wise regression tasks [30]. FDN Net uses KL-divergence instead of cross-entropy loss to train models [31]. Ordinal loss has also been used by some researchers. These methods essentially leverage soft classification tasks to deal with the HPE task [32] due to the systematic errors that are caused by inaccurate labels and the random errors that are caused by subject diversity within HPE. Some researchers have also considered using quaternions [33] and rotation matrices [34] instead of directly regressing to PYR. However, these methods tend to amplify errors during transformations. After analyzing these existing methods, we developed a heterogeneous loss to train our model, which could deal with the continuity problem by transforming it into a classification problem and constraining the feature spaces.

### 2.2. Visual Sensor-Based 3D Gaze Estimation

Gaze direction estimation involves two kinds of approaches: geometry-based and appearance-based methods. The concept of geometry-based methods is the use of feature positions to calculate gaze through geometric relationships, which requires a high resolution and high image quality. With advances in deep learning, appearance-based methods have been increasingly adopted because they are robust to low-quality images.

The classic approach uses a single eye image as the input. To improve performance, some researchers have also used head pose to enhance the input. However, some experiments have found that the benefits of using head pose are very limited [35] because head pose estimation is not accurate. To avoid this problem, some researchers have leveraged facial images instead of head pose [36]. However, facial images are usually more diverse than eye images, which affects the robustness of person independence. Some researchers have also explored the asymmetry between the two eyes [37]. However, these studies focused on asymmetry and neglected the basic feature of the two eyes, i.e., consistency. The consistency between the two eyes was explored in this study and significantly improved the performance of our model.

The aforementioned studies focused on person-independent methods, which meant that the training samples and test samples were from different subjects. Considerable research has focused on personalized methods [38], such as the use of training and test samples from the same subject to explore personalized bias. A common method is to use a few labeled samples from test subjects to calibrate the model [39], which can significantly improve the accuracy of the model. Some researchers have also explored the use of unsupervised and semi-supervised methods to handle this task [40]. This study focused on the person-independent model; however, calibration was also discussed.

## 3. Methodology

**Figure 2:**
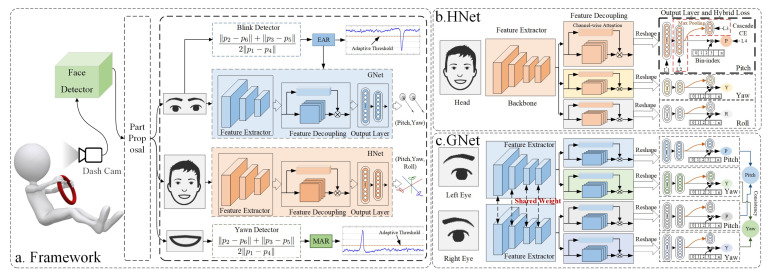
(**a**) The architecture of the proposed appearance-based multi-state driver monitoring system; (**b**) the structure of HNet, which was used for head pose estimation; (**c**) the structure of GNet, which was used for gaze estimation. A demonstration video can be found at https://www.youtube.com/watch?v=fS4jSiZYGUU, accessed on 22 March 2022.

This study developed an appearance-based framework for multi-state driver monitoring. The driver’s face was detected by a face detector. Then, the detected bounding boxes and landmarks were used to allocate different regions for specific tasks. Blinking and yawning behaviors were measured using the eye aspect ratio (EAR) and the mouth aspect ratio (MAR), respectively. To solve the challenge of HPE and GE, this study used HNet and GNet, respectively. The architecture is shown in Figure 2. HNet leveraged a pre-trained model as the feature extractor and an FDM, which was designed to decouple the features from different branches. During training, a heterogeneous loss was used. GNet was similar to HNet, but the left and right eyes shared the same weights for the feature extractor. The estimation of the left and right eyes was optimized using the proposed principle of gaze consistency.

### 3.1. Attention-Based Feature Decoupling

The outputs for the head pose and gaze estimations were similar in form, which represented the angles on different axes. We considered that the head pose estimation model could obtain the probability P(Y|X), where $X={\left\{x^{i}\in R^{3\times H\times W}\right\}}^{N}$ and $Y={\left\{y^{i}\in R^{3}\right\}}^{N}$. The common approaches directly calculate the joint probability $P(Y_{pyr}|X)$, but when $Y_{pyr}$ was decoupled, the model could be converted to calculate the different marginal probabilities separately, as follows:

(1) $$P\left(Y_{pyr}|X\right),s.t.Y\in R^{3}\to \left\{\begin{array}{c} P\left(Y_{p}|X\right),s.t.Y_{p}\in R\\ P\left(Y_{y}|X\right),s.t.Y_{y}\in R\\ P\left(Y_{r}|X\right),s.t.Y_{r}\in R \end{array}\right.$$

which reduced the complexity and the required number of training samples.

To achieve this goal, a feature decoupling module leveraging an attention mechanism was designed. Convolutional attention modules can basically be divided into two categories: channel and spatial attention modules. Spatial attention modules allow models to learn to focus on specific spatial locations by utilizing the inter-spatial relationships between features. Channel attention modules allow models to pay attention to important channels by exploiting the inter-channel relationships between features. In this study, an attention module was used after the feature extractor. Therefore, it allowed our model to focus on different feature maps for each angle. Spatial-wise attention was not used because it is equivalent to channel attention after averaging pooling. To force the model to decouple the feature vector of each angle, there was no skip connection in the attention module. The channel attention module that we used was computed as follows:

(2) $$\begin{array}{cc} F & =\sigma (MLP(AvgPool(F)))+MLP(MaxPool(F))\\ \; & =\sigma \left(W_{1}\left(W_{0}\left(F_{avg}^{c}\right)\right)\right)+W_{1}\left(W_{0}\left(F_{max}^{c}\right)\right) \end{array}$$

where σ represents the sigmoid function and $W_{0}\in R^{C/r\times C}$ and $W_{1}\in R^{C\times C/r}$ are the weights of the *MLP*, which were shared for both input feature maps.

### 3.2. Heterogeneous Loss with Cascade Cross-Entropy

This study developed a maintainable, upgradeable, and flexible framework based on a series of pre-trained models. Typically, these models are trained for classification tasks. To maximize their learning ability, this study converted HPE and GE from regression tasks into classification tasks. However, there were several challenges to overcome. The first was that continuous output had to become a discrete output. One approach to achieve this could be to use a fine-grained class as owing to the inherent noise and errors in data, fine-grained classes are effective. Aggregation and hierarchy could also be used to improve performance through similar bagging strategies, which can average multiple outputs. Another approach could be to calculate a weighted average across multiple output classes, which is divided into several bins, as follows:

(3) $$\theta_{pred}=\omega *\left(\sum_{i=0}^{M}\rho_{i}*i-\frac{M}{2}\right)+\frac{\omega }{2}$$

where *M* is the number of bins, ω is the width of each bin, and $\rho_{i}$ is the corresponding probability of the *i*th bin. The model could then obtain a continuous output from the weighted average, which could solve the continuity problem; however, another challenge was the correlation problem. The most widely used loss function for classification tasks is cross-entropy (CE); however, the problem with CE is that it is the same between adjacent classes and other classes. It is reasonable to use for normal classification tasks, but not for HPE and GE. To overcome these challenges, a cascade cross-entropy loss function was developed, which was used to aggregate the heterogeneous loss, as follows:

(4) $$\begin{array}{cc} L & =L_{cce}+\lambda_{3}L_{reg}+\lambda_{4}L_{cent}\\ \; & =\left(\lambda_{1}L_{ce}+\lambda_{2}L_{ce_{\phi }}\right)+\lambda_{3}L_{reg}+\lambda_{4}L_{cent}\\ \; & =\lambda_{1}\frac{1}{N}\sum_{i}-\sum_{c=0}^{M}y_{ic}log\left(\rho_{ic}\right)+\lambda_{2}\frac{1}{N}\sum_{i}-\sum_{c=0}^{M/b}\hat{y_{ic}}log\left(\phi \left(\rho_{ic}\right)\right)\\ \; & +\lambda_{3}\frac{1}{2N}\sum_{i}{∥\theta_{gt}-\theta_{pred}∥}^{2}+\lambda_{4}\frac{1}{2N}\sum_{i}{∥x_{i}-c_{y_{i}}∥}_{2}^{2} \end{array}$$

where $L_{cce}$ is the cascade cross-entropy loss (including $L_{ce}$ and $L_{ce_{\phi }}$), $L_{ce}$ is the standard cross-entropy loss, $L_{ce_{\phi }}$ is the term used to penalize predictions that were far away from the ground truth via the ϕ(·) function that maximized the original output, *b* is the corresponding step number, $y_{ic}$ denotes the label of the *c*th class of the *i*th sample, $L_{reg}$ is the mean squared error of the corresponding weighted average (based on Equation (3)), $\theta_{gt}$ is the ground truth, $L_{cent}$ is the center loss (which was used to minimize intra-class variations while keeping the features of different classes separate), $x_{i}$ is the *i*th extracted feature, and $c_{y_{i}}$ denotes the $y_{i}$th class center of the corresponding features (which was updated as deep features changed). The output was a coarse-grained output compared to the original fine-grained output. Finally, the hyper-parameter $\lambda_{1,2,3,4}$ was used to balance the different losses.

### 3.3. Eye Consistency Principle

GE is basically the same as HPE and similar models can be used. However, the difference is that GE can use two eye images instead of a single side image. Some researchers have even used facial images to provide more information and improve performance. Ref. [37] explored the asymmetry between eyes that allows them to complement each other under certain unfavorable conditions. However, the authors ignored an important feature of eyes: consistency. The gaze directions of the left eye and right eye are usually estimated separately or an MLP layer is used to fuse the features of the two eyes to obtain an overall gaze direction.

**Figure 3:**
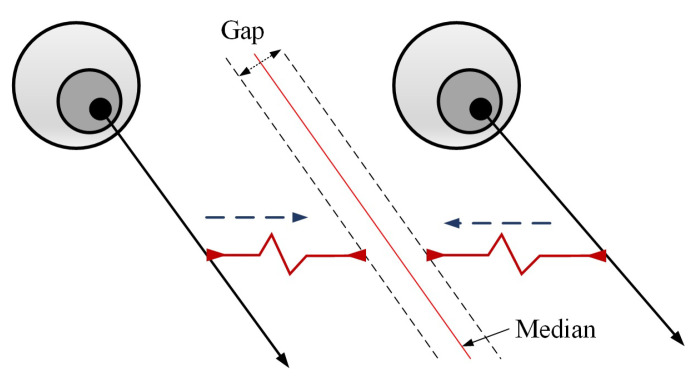
The principle of gaze consistency (the gap is usually small).

By analyzing datasets and real user experiences, it has been found that the gaze directions of the left eye and right eye are basically the same under natural conditions. In some experimental environments, there has been a small difference in the yaw direction between the two eyes, but they still followed the principle of consistency as a whole. To leverage this principle, GNet was designed, as shown in Figure 2. The feature extractors of the eyes were the same and they shared the same weights. Moreover, they independently estimated gaze direction using the decoupling module and the output module. The key step was that the estimation of the gaze directions of the left and right eyes was adjusted according to the principle of consistency, as shown in Figure 3.

(5) $$\left\{\begin{array}{c} p_{l}=\lambda_{l}*p_{l}+\left(1-\lambda_{l}\right)*p_{r}+0.5*\varepsilon \\ p_{r}=\lambda_{r}*p_{r}+\left(1-\lambda_{r}\right)*p_{l}-0.5*\varepsilon \end{array}\right.$$

where λ denotes the weight and ε denotes the average gap between the left and right eyes. The principle of consistency significantly improved the accuracy of the estimation, similar to the idea of ensemble learning, which leverages multiple independent models to reduce the likelihood of the selection of a poor model.

### 3.4. Blinking and Yawning Detection Using Aspect Ratios

Existing blinking and yawning detection methods use either landmark-free or landmark-based approaches. Landmark-free methods usually require specific models to detect blinking or yawning. Ref. [41] proposed a real-time blinking detection method using facial landmarks and defined the eye aspect ratio (EAR) that determines whether an eye is open or closed as the following:

(6) $$\mathrm{EAR}=\frac{∥p_{2}-p_{6}∥+∥p_{3}-p_{5}∥}{2∥p_{1}-p_{4}∥}$$

where $p_{1},\;\ldots \;,p_{6}$ denote the different landmarks of an eye that can be obtained from a face detector. State-of-the-art landmark detectors are robust for various illumination conditions, facial expressions, and non-frontal head rotations. In this study, Dlib [42] was utilized as the face detector, which is a popular library and is simple to use. Owing to the fact that a face detector was required for both head pose and gaze estimation, this study adopted EAR to detect blinking in order to reduce extra calculations, as well as adopting the mouth aspect ratio (MAR) for yawning detection. The thresholds were obtained from subject-wise calibration.

## 4. Experiments

### 4.1. Datasets

**Figure 4:**
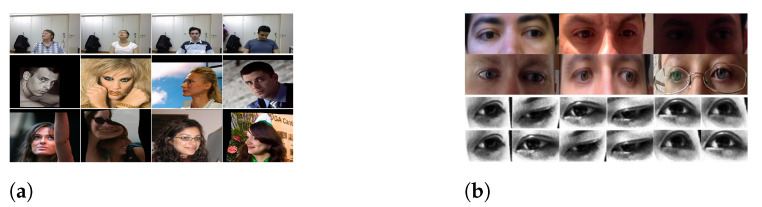
(**a**) Images from the head pose datasets: BIWI (first row), AFLW2000 (second row), and 300W-LP (third row); (**b**) images from the gaze datasets: MPIIGaze (top) and UT-Multiview (bottom).

Currently, the most popular datasets for HPE are 300W-LP [43], AFLW2000 [43], and BIWI [44], as shown in Figure 4a. 300W-LP provides accurate head poses and additionally generates synthetic views to expand its library to 122,450 samples. AFLW2000 provides accurate head poses for the first 2000 images of the AFLW dataset. It exhibits large pose variations for various illumination conditions and expressions. BIWI contains over 15,000 images of 20 people. For each frame, a depth image, the corresponding RGB image, and annotation are provided.

There are two widely used datasets for GE: MPIIGaze [35] and UT-Multiview [45], as shown in Figure 4b. MPIIGaze is an appearance-based gaze dataset that includes 15 participants and has a large variability in appearance and illumination conditions. Only 1500 images of the left and right eyes of the subjects were randomly selected for training and testing. UT-Multiview contains images of a total of 50 people who participated in the data collection, which cover 160 different gaze directions that were acquired using eight cameras. In total, it includes 64,000 real eye images and 1,152,000 synthesized eye images.

### 4.2. Experimental Protocol

HPE: The widely used protocol for HPE is to train on the synthetic 300W-LP dataset and test on the two real-world datasets (AFLW2000 and BIWI). The 300W-LP and AFLW2000 datasets provide facial landmarks that can be used to loosely crop the head area, whereas the BIWI dataset uses a face detector to obtain the head images. The head pose range was set to [−99,+99], which was the same setting as in [28,30].

GE: We followed the protocols that were used in the original paper and by other researchers. MPIIGaze applies the leave-one-subject-out protocol, whereas the UT-Multiview dataset uses the 3-fold cross-validation protocol. Only real samples were tested, which was also the same as in [39].

Baseline: To evaluate the proposed method, two baseline principles were used for HPE:

1. Two fully connected layers were used to estimate the features that were extracted from the backbone feature extractor and the number of hidden layers was 512;
2. Only $L_{ce}$ and $L_{reg}$ were used to train the model without feature decoupling. One baseline principle was used for GE, which was similar to the first baseline principle for HPE, but both the left and right eyes were used as the input.

Evaluation Metrics: The mean absolute error (MAE) metric was used to quantify the head pose estimation error:

(7) $$\mathrm{MAE}=\frac{1}{N}\sum_{i=1}^{N}\left(\left|\hat{p_{i}}-p_{i}\right|\right)$$

where *N* is the number of test datasets and $\hat{p_{i}}$ and $p_{i}$ represent the ground truth and prediction, respectively.

The angular error is widely used to measure the accuracy of 3D gaze estimation:

(8) $$L_{ang}=arccos\left(\frac{g\cdot \hat{g}}{∥g∥∥\hat{g}∥}\right)$$

where g∈R is the predicted gaze vector and $\hat{g}$ is the ground truth.

### 4.3. Experimental Results

**Figure 5:**
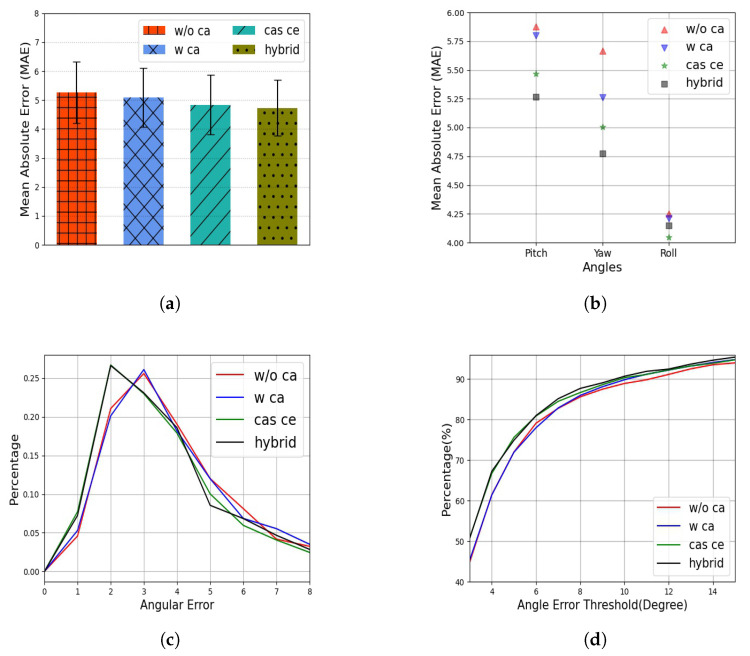
(**a**) The mean absolute error of the different paradigms on the AFLW2000 dataset; (**b**) the mean absolute error of the different paradigms on the different axes; (**c**) the percentage of different errors using the different paradigms; (**d**) the percentages of correct samples of the different paradigms under different thresholds.

**Figure 6:**
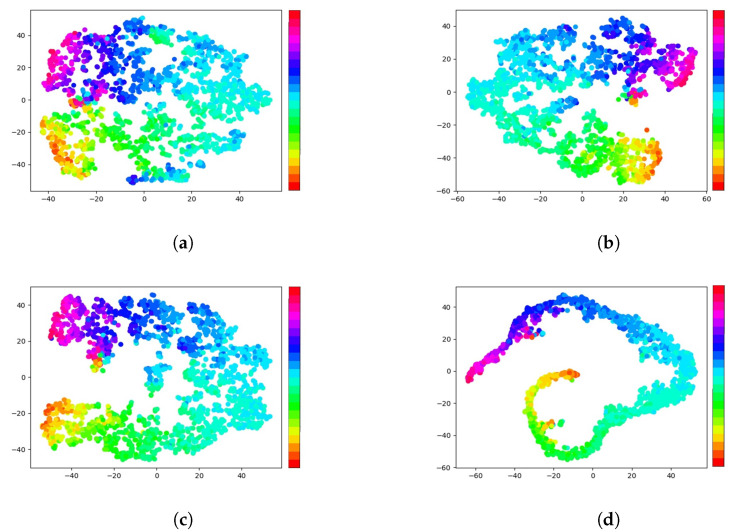
The tSNE visualization of the extracted features from the different paradigm models. The samples were divided into several bins with different colors, according to their yaw angles: (**a**) w/o ca; (**b**) w ca; (**c**) cas ce; (**d**) hybrid.

To comprehensively verify the proposed method, four models were trained using different paradigms: *w/o ca*,*w ca*, *cas ce*, and *hybrid*. The *w/o ca* paradigm was the same as *Baseline 2* without feature decoupling, whereas *w ca* included the feature decoupling module, *cas ce* used CasCE for training, and *hybrid* was the proposed method, which included feature decoupling and a heterogeneous loss function. In this experiment, EfficientNet-b0 was chosen as the backbone model. The values of the hyper-parameters $\lambda_{1}$, $\lambda_{2}$, $\lambda_{3}$, and $\lambda_{4}$ were 0.5, 1.0, 2.0, and 0.01, respectively. The results of the experiment are presented in Figure 5. It can be seen that the proposed method decreased the mean absolute error and improved the overall performance. Usually, the angle range was wider on the yaw axis and the effect of the angle was more obvious on the yaw axis, as shown in Figure 5b. Correspondingly, the difference was small on the roll axis, which had a small angle range. This was one of the reasons why the MAE on the roll axis was lower than those on the other axes. Notably, the CasCE paradigm decreased the error on all three axes. The purpose of the CasCE paradigm was to restrict larger errors, which was verified, as shown in Figure 5c,d. The small percentage of errors from the *cas ce* and *hybrid* methods were significantly larger than those of the other two paradigms. To further explore the proposed method, the features that were extracted by the four paradigms were visualized using the tSNE algorithm, as shown in Figure 6. To facilitate our observations, samples from the AFLW2000 dataset were divided into 22 bins, according to their yaw angles. The various colors in the figure represent the different bins, as shown by the color bar. A large distance between two colors represents a large difference in yaw angle. It can be seen that the proposed method increased the discrimination of different bins, especially those that had a large difference in yaw angle.

**Figure 7:**
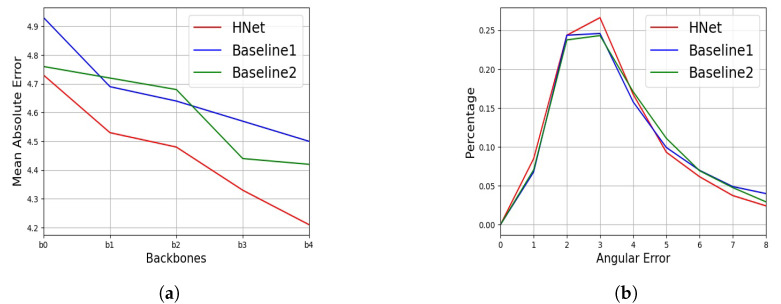
The mean absolute error and percentage of angular errors on the AFLW2000 dataset: (**a**) the mean absolute error when different versions of EfficientNet (b0–b4) were used as the feature extractor; (**b**) the percentage of angular errors using EfficientNet-b4.

**Figure 8:**
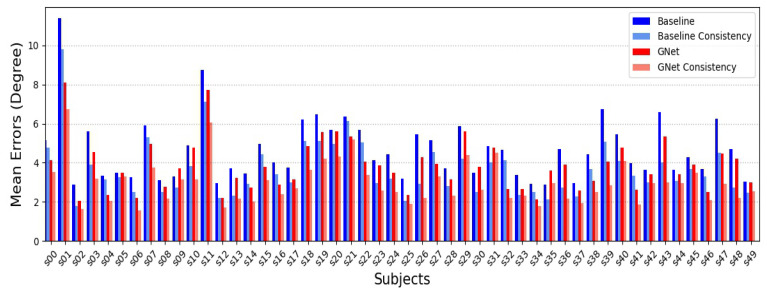
A comparison between the results from the baseline model and the proposed GNet model, both with and without the gaze consistency principle, for different subjects from the UT-Multiview dataset.

**Figure 9:**
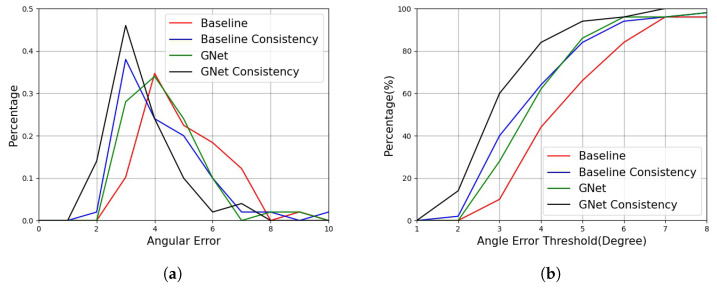
The gaze error distribution on the UT-Multiview dataset: (**a**) the percentage of different errors; (**b**) the percentage of correct samples under different thresholds.

To verify the influence of the backbone model, the proposed HNet was compared to the two baseline methods using different EfficientNet backbone models on the AFLW2000 dataset. The results are shown in Figure 7. It can be seen that the proposed method significantly reduced angular errors and that the two baseline methods were essentially the same. The proposed method also suppressed large errors, as verified by the percentage of angular errors, as shown in Figure 7b. Note that both baseline methods achieved a better performance than many other methods, which demonstrated that the backbone model was also important for the methods and that the proposed framework achieved good maintainability and upgradability. This was also verified for the GE task, as shown in Figure 8 and Figure 9. The proposed GNet model decreased the mean gaze error and increased the percentage of lower gaze error compared to the baseline model. These experiments demonstrated that the proposed method was effective.

For the GE task, this study introduced the gaze consistency principle. To verify this, comparative experiments were conducted, the results from which are shown in Figure 8 and Figure 9. The values of $\lambda_{l}$ and $\lambda_{r}$ were 0.5, whereas ε was 0. The principle of consistency was used in both the baseline and GNet models. The results showed that the proposed principle significantly decreased gaze errors and the improvement effect even exceeded the benefits of the proposed model. The reason for this was the GNet model was similar to simple ensemble learning models that combine two independent models (left eye and right eye) to reduce the likelihood.

**Figure 10:**
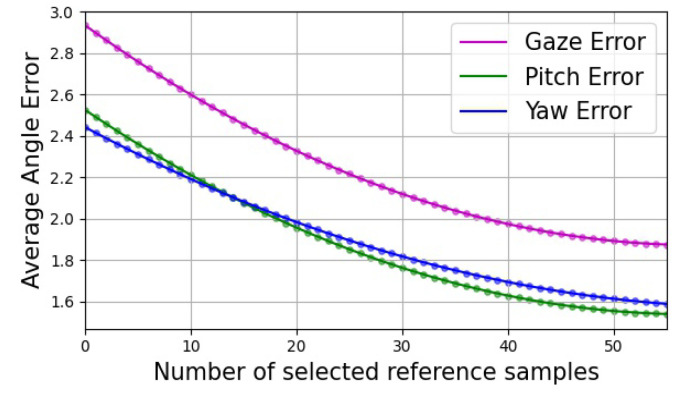
The angle errors using different numbers of reference images from the UT-Multiview dataset.

It can be seen that the error variance between different subjects was very large, as shown in Figure 8. To eliminate this subject-wise bias, some researchers have proposed reference-based methods, but the most common approach is to calibrate the model using a few samples. To verify whether the proposed method could be fine-tuned, different numbers of reference samples were used to calibrate the trained GNet model, as shown in Figure 10. This calibration significantly reduced the gaze errors, based on a few reference samples.

### 4.4. Comparison to State-of-the-Art Methods

To further evaluate our proposed method, we compared it to the best published methods in the literature.

#### 4.4.1. Head Pose Estimation

HPE can be addressed using landmark-free or landmark-based learning. The landmark-free methods mainly focus on the output format design and explore various representations of rotation. The results from landmark-free methods are shown in the upper part of Table 1. Owing to the existence of labeling errors and other reasons, accuracy is becoming increasingly difficult to improve. Recently, landmark-based models have shown their effects and significantly reduced errors, as shown in the middle part of Table 1. However, it is worth noting that they usually modify the training and test datasets. MNN [27] used a modified 300W-LP dataset to train the model rather than the original head pose labels. The img2pose [22] approach manually annotated 68-point ground truth landmarks in a test dataset instead of the original incorrect 21-point landmarks. This preprocessing helped the model to take advantage of facial landmarks to improve its performance. We trained the proposed model using a series of backbone models and the results are shown in the bottom part of Table 1. On the AFLW2000 dataset, our method had the lowest errors in the landmark-free group and it could also compete with landmark-based methods. On the BIWI dataset, our method achieved the lowest errors in both groups. This demonstrated that our method outperformed other state-of-the-art methods for the HPE task.

#### 4.4.2. Gaze Estimation

As mentioned above, there are two kinds of approaches to the GE task: reference-free (nRef) and reference-based (Ref) approaches. The reference-based methods are similar to calibrated models, which require several reference samples from a calibration dataset. For comparison, we calibrated the trained model using different numbers of reference samples. To improve the performance of models, some researchers have used additional information, including facial images or head poses. However, only eye images were used to validate our methods. In the GE experiment, only EfficientNet-b0 was used as the backbone model. The results are shown in Table 2. Our method achieved a state-of-the-art performance on both the MPIIGaze dataset and the UT-Multiview dataset, although the performance on the latter dataset was better than that on the former. This was because the latter dataset was larger than the former. The leave-one-subject-out protocol also had more subject bias than the 3-fold cross-validation protocol. After calibration using a few reference samples, the performance of the models significantly improved. These results demonstrated that the proposed method was feasible and efficient.

## 5. Conclusions

To monitor the facial states of drivers, this paper proposed an appearance-based framework that included head pose estimation, gaze estimation, and blinking and yawning detection. This study mainly focused on the former two challenging tasks. To preserve the maintainability and upgradability of the framework, we adopted a pre-trained backbone model as the feature extractor. After analyzing the tasks, a feature decoupling module was first developed to obtain the features of the different axes. Then, a cascade cross-entropy was designed to restrict large errors. Finally, a heterogeneous loss function was built to optimize the training process. Experiments were conducted and the results demonstrated that the proposed approach could improve feature representation discrimination, as well as model accuracy. Moreover, this study used the gaze consistency principle to improve gaze estimation performance. To compare its performance to that of other methods, the proposed approach was evaluated on two popular benchmark datasets for each task. The comparison results demonstrated that the proposed approach could achieve a state-of-the-art performance while maintaining the highest accuracy.

## Figures and Tables

**Table 1:** A comparison of head pose estimation results.

Method	AFLW2000				BIWI			
	Pitch	Yaw	Roll	Mean	Pitch	Yaw	Roll	Mean
KEPLER [29]	-	-	-	-	17.2	8.08	16.1	13.8
Dlib(68) [30]	13.6	23.1	10.5	15.8	13.8	16.8	6.19	12.2
FAN(12) [29]	7.48	8.53	7.63	7.88	-	-	-	-
3DDFA [43]	8.53	5.40	8.25	7.39	12.3	36.2	8.78	19.1
HPE-40 [46]	6.18	4.87	4.80	5.28	5.18	4.57	3.12	4.29
HopeNet [28]	6.55	6.47	5.43	6.15	6.60	4.81	3.26	4.89
Shao [29]	6.37	5.07	4.99	5.48	7.25	4.59	6.15	6.00
SSR-Net [30]	7.09	5.14	5.89	6.01	6.31	4.49	3.61	4.65
FSA-Net [30]	6.08	4.50	4.64	5.07	4.96	4.27	2.76	4.00
QuatNet [33]	5.61	3.97	3.92	4.50	5.49	4.01	2.93	4.14
FDN [31]	5.61	3.97	3.88	4.42	4.70	4.52	2.56	3.93
WHENet-V [29]	5.75	4.44	4.31	4.83	4.10	3.60	2.73	3.48
TriNet [34]	5.76	4.19	4.04	4.66	4.75	3.04	4.11	3.97
Ordinal [32]	-	-	-	-	4.36	3.68	3.02	3.69
DGDL [46]	5.35	3.77	4.06	4.39	4.46	3.63	3.08	3.72
MNN [27] †	4.69	3.34	3.48	3.83	4.61	3.98	2.39	3.66
img2pose [22] ‡	5.03	3.42	3.27	3.91	3.54	4.56	3.24	3.78
**HNet-b0**	5.24	4.47	4.05	4.58	4.74	3.56	3.22	3.84
**HNet-b1**	5.17	4.41	3.68	4.42	4.43	3.20	3.20	3.61
**HNet-b2**	5.03	4.28	3.71	4.34	4.79	3.12	2.95	3.62
**HNet-b3**	5.04	3.98	3.55	4.19	3.86	3.30	3.12	**3.42**
**HNet-b4**	4.70	4.07	3.20	**3.99**	4.35	3.46	3.30	3.70

† denotes that the model was not trained using the original dataset; ‡ denotes that the model re-annotated the test dataset. The bold aims to highlight our proposed methods, as well as the best results.

**Table 2:** A comparison of gaze estimation results.

Method		Input		MPIIGaze			UT-Multiview		
		Face	Head	Center	Right	Avg	Center	Right	Avg
nRef	iTracker [47]		-	5.6	5.6	5.6	-	-	-
	GazeNet [36]	-	✓	5.5	5.5	5.5	4.4	4.4	4.4
	Dilated-Net [47]	✓	-	5.2	5.2	5.2	-	-	-
	CrtCLGM [48]	✓	-	-	-	-	5.7	5.7	5.7
	MeNet [47]	✓	-	4.9	4.9	4.9	5.5	5.5	5.5
	RT-GENE [47]	✓	-	4.8	4.8	4.8	5.1	5.1	5.1
	LNSMM [49]	-	-	4.8	4.8	4.8	4.8	4.8	4.8
	U-Train [40]	-	-	-	-	-	5.5	5.5	5.5
	GEDDne [50]	✓	-	4.5	4.5	4.5	-	-	-
	PureGaze [51]	✓	-	4.5	4.5	4.5	-	-	-
	BAL-Net [52]	-	-	4.3	4.3	4.3	5.4	5.4	5.4
	FAR-Net [37]	✓	-	4.3	4.3	4.3	-	-	-
	I2D-Net [47]	✓	-	4.3	4.3	4.3	-	-	-
	AGENet [47]	✓	-	4.1	4.1	4.1	-	-	-
	CA-Net [51]	✓	✓	4.1	4.1	4.1	-	-	-
	** GNet-b0**	-	-	3.83	4.01	**3.92**	2.97	2.98	**2.98**
Ref	DEANet [53]	-	✓	4.38	4.38	4.38	3.56	3.56	3.56
	Diff-NN [39]	-	-	4.69	4.62	4.64	4.17	4.08	4.13
	Diff-VGG [39]	-	-	3.88	3.73	3.80	3.88	3.68	3.78
	**GNet-b0(5)**	-	-	3.13	3.28	**3.21**	2.74	2.75	**2.75**
	**GNet-b0(10)**	-	-	3.19	3.17	**3.18**	2.60	2.59	**2.60**
	**GNet-b0(20)**	-	-	2.98	3.04	**3.01**	2.33	2.33	**2.33**

The bold aims to highlight our proposed methods, as well as the best results.

## Data Availability

Not applicable.
